# Drawing on Strategic Management Approaches to Inform Nutrition Policy Design: An Applied Policy Analysis for Salt Reduction in Packaged Foods

**DOI:** 10.34172/ijhpm.2020.204

**Published:** 2020-11-01

**Authors:** Helen Trevena, Bruce Neal, Shauna M. Downs, Teresa Davis, Gary Sacks, Michelle Crino, Anne Marie Thow

**Affiliations:** ^1^Menzies Centre for Health Policy, University of Sydney School of Public Health, Sydney, NSW, Australia.; ^2^Food Policy Division, The George Institute for Global Health, The University of New South Wales, Sydney, NSW, Australia.; ^3^The School of Public Health, Faculty of Medicine, Epidemiology and Biostatistics, Imperial College of Science, Technology and Medicine, London, UK.; ^4^Charles Perkins Centre, University of Sydney, Sydney, NSW, Australia.; ^5^The Royal Prince Alfred Hospital, Sydney, NSW, Australia.; ^6^Department of Urban-Global Public Health, Rutgers School of Public Health, Newark, NJ, USA.; ^7^Discipline of Marketing, University of Sydney Business School, Sydney, NSW, Australia.; ^8^Global Obesity Centre, Deakin University, Geelong, VIC, Australia.

**Keywords:** Salt Reduction, Food System Drivers, Australia, Nutrition Policy

## Abstract

**Background:** Nutrition policies to improve the food environment frequently rely on voluntary business action for implementation, many have had mixed success. The aims of this study were to identify key food system drivers influencing the Australian packaged food sector and analyse how these might impact the willingness of food companies to voluntarily reduce salt in packaged foods.

**Methods:** Business methods formed the basis of this retrospective applied policy analysis of voluntary salt reduction for the period 2013-2016 where the focal policy was the Australian Food and Health Dialogue (2009-2015). The analytical framework included political-legal, economic, social, technological (PEST) external drivers of the food system, and Porter’s Five Forces for the competitive drivers of the food system. Documentary data identifying food system drivers affecting the Australian packaged food sector (comprised of the food processing and supermarket industries) were identified through a comprehensive search of the grey and academic literatures.

**Results:** The interplay between external and competitive food system drivers created an environment in which voluntary salt reduction was found to be an uneasy fit. A high cost of doing business, soft growth, intense competition, asymmetry of power in favour of supermarkets, and marginal consumer interest in less salty food were found likely to create commercial disincentives to invest in voluntary salt reduction above more pressing commercial imperatives. Analysis of food manufacturing industries highlighted the highly contextual nature of food system drivers. Opportunities for nutrition policy included: support for ‘shared value’ in economic discourse; and, leveraging investor, supermarket, and the largely unrealised bargaining power of consumers.

**Conclusion:** Business frameworks can provide meaningful insights for nutrition policy on how food system drivers can thwart policy goals. Our analysis highlighted areas to incentivise voluntary action and illustrated the importance of political-legal, economic and consumer strategies for salt reduction.

## Background

Diet is a leading contributor to the growing global burden of non-communicable disease, which includes diabetes, some cancers, and cardiovascular disease (CVD).^1,2^ These diseases are largely attributable to the unfavourable metabolic effects from foods and drinks containing excess saturated fats, sugar, energy, and salt.^3^ Governments globally have committed to taking action on diets and nutrition. Nutrition is inextricably linked to countries achieving the Sustainable Development Goals (SDGs); goals which are universal, apply to all countries and were officially adopted by 193 Member States of the United Nations.^4^ SDG 3 is to ensure healthy lives and promote well-being for all at all ages and target 3.4 is to reduce premature mortality from non-communicable disease by one third by 2030.^4^ One of many strategies to achieve this is improving nutrition by creating healthier food environments. However, unhealthy food environments persist in all countries, characterised by the promotion, marketing and easy availability and affordability of foods containing levels of salt, fat, and sugar considered too high for a healthy diet.^5,6^

Nutrition policies aim to enable healthier choices through the creation of healthier food environments in areas such as nutrition labelling, responsible marketing, and product quality (eg, product safety and formulation/reformulation).^7^ For the purposes of this paper we define policy as a broad statement of goals, objectives and a way to create a framework for policy action.^8^ Many public policies are predicated on the voluntary actions of business for implementation^9^ – regarded as providing some opportunities but carrying more risk^10^ and less effective than mandated approaches.^11^ Industry influence and/or led rules and standards, and weak public-partnerships are often cited reasons for poor policy outcome.^10,12-17^ For example, the comprehensive United Kingdom salt reduction campaign was widely regarded as the benchmark other countries sought to emulate, but lost momentum when the responsibility for salt reduction passed to food companies with the Responsibility Deal in 2011.^18^ Australia adopted a similar policy mechanism with the Australian Food and Health Dialogue (Dialogue)^19^ (ie, food companies voluntarily agreed to reduce added salt).

The Dialogue^19^ was established in 2009, led by the Department of Health it also included members from non-government organisations, government agencies, and the private sector. Whilst there were concurrent long-standing non-governmental advocacy campaigns,^20^ domestic^21^and global^22,23^ corporate initiatives, the Dialogue was the focal point for salt reduction during this study period (2013-2016). Salt reduction targets were in place for selected packaged products from 9 food categories, chosen because of their sizeable contribution to salt intake from packaged food in Australia. The targets for breads, ready-to-eat breakfast cereals, and processed meats were to be achieved by December 2013; simmer sauces, soups and savoury pies by December 2014; potato/corn/extruded snacks, and savoury crackers by December 2015; and cheese by December 2017.^19^ (See Supplementary file 1 for further detail of the Dialogue salt reduction targets). The Dialogue had modest success in achieving some of the targets^16,17,24^ and after a protracted period of inactivity it was replaced in November 2015 by the currently active Healthy Food Partnership.^25^ New salt reduction targets were proposed by the Healthy Food Partnership in 2018 for implementation starting July 2020.^26^ It remains to be seen if these will be implemented more successfully than those of the Dialogue.

This study takes a new approach to analysing why nutrition policies predicated on voluntary action have largely failed to make meaningful improvements in food environments. It focuses on the external political-legal, economic, social, technological (PEST), and competitive food system drivers and how they may affect business behaviour to act on voluntary nutrition policies in Australia. Understanding food system drivers and how they function is increasingly regarded as a necessary first step towards more effective nutrition policy.^27^ In this study the food system is described as the elements (environment, people, inputs, processes, infrastructures, institutions) and activities that relate to the production, processing, distribution, preparation and consumption of food, and the output of these, including the socio-economic and environmental outcomes.^28^ As would be expected in a complex, adaptive system involving interactions by many actors and institutions,^29^ food systems are profoundly shaped by many drivers depending on the boundaries of the system.^27^ Understanding these drivers is acutely pressing for nutrition policies premised on business goodwill for successful implementation, and is thus especially pertinent for voluntary reformulation, such as salt reduction.

In contrast to public health, business has long-recognised the importance of identifying and assessing the impact of ‘drivers of their system.’ Mindful of a particular ‘sector’ – large segments of the economy in which a large number of companies operate – and ‘industry’ – where a specific group of companies or businesses operate in a similar area^30^- an external and competitive analysis seeks to identify and assess the business implications of the current and predicted external PEST^31^ and competitive drivers^32^ to inform business decision-making.^33^ While understanding competitive patterns within a sector (eg, Australian packaged food sector that in this study incorporates food processing/manufacturing and retailing) or industry (eg, supermarket industry, cheese food manufacturing industry) may seem foreign to public health actors, a comprehensive assessment of competitive strength can highlight the distribution of power, as well as attractiveness to invest in, and the potential implications for, the innovation of healthier products.^31^ As the biggest investors in the food system,^34^ the decisions made by business on the availability, price, product quality, promotion and marketing of food heavily influence the food environment.^35,36^ Voluntary action to implement nutrition policy is just one of the many considerations for a business alongside meeting their on-going economic and legal obligations. To the best of our knowledge, few studies have taken an interdisciplinary approach to identifying external and competitive food system drivers and assessing how they might function to mediate the implementation of nutrition policy that is voluntary such as the Dialogue.

In this study, we adapt established frameworks from strategic management to analyse the external and competitive drivers of food industry decision-making, using the case study of voluntary salt reduction in Australia in which the focal policy was the Dialogue.^19^ Salt reduction is a global health policy priority, due to the dose response relationship between salt intake and blood pressure in adults, which is a leading risk factor for CVD.^37^ A 30% relative reduction in mean population intake by 2025 was endorsed by Member States of the World Health Organization (WHO), including Australia.^38^ Despite being a key policy intervention to prevent CVD, salt reduction of the scale and magnitude needed to save lives remains challenging.^39,40^ The aims of this study were to identify the key food system drivers influencing the Australian packaged food sector and analyse how these might impact the willingness of food companies to voluntarily reduce salt in packaged foods, and to identify any implications for similar voluntary nutrition policies.

## Key Messages

**Implications for policy makers**

- There is a growing awareness of the importance to better understand food system drivers and their interactions in nutrition policy. Analysis of these drivers can serve to highlight how food system drivers could be harnessed to influence business behaviour in support of effective nutrition policy.
- There is an opportunity for nutrition policy-makers to leverage off consumer health trends in the design of nutrition policy.
- Competitive advantage requires a business to provide products that meet consumer needs better than their competitors, incorporating a citizen science approach could strengthen policy-makers understanding of the public perspective of the food environment.
- To lessen the threat of product substitution with unhealthier alternatives and promote a level playing field for business, policy-makers could work towards nutrition policy coherence across the packaged food and food service sectors.

**Implications for public**

Food businesses are the biggest investor in the food system and the decisions they make on product quality, price, promotion and where products are sold influences what, where, why and how we buy, prepare and eat food. Many packaged foods contain excess salt, sugar and fat. Different business decisions about the foods we eat have the potential to help the public to be healthier. This research has highlighted the tremendous unrealised bargaining power of the public. Unleashing this power could be achieved through every-day purchase decisions and by participating in initiatives to improve the food environment. These initiatives could include applying pressure to incorporate nutrition considerations into investment decision-making, and participation in citizen science approaches to explore the public experience of their food environment to help design nutrition policy.

## Methods

###  Study Design and Framework

The present study is a retrospective applied policy analysis informed by business analysis (strategic management) approaches and frameworks, focused on salt reduction. The research questions were: ‘ *what are the key external PEST, and competitive food system drivers of the Australian packaged food sector*?’ and ‘ *how might they function to affect the willingness of a food company (business) to implement salt reduction policy that is voluntary*?’ We conceptualised the main groupings of food system drivers as the drivers of change used by business to conduct an external and competitive analysis. We illustrate the context specificity of food system drivers using data relevant to selected food manufacturing and supermarket industries. The study design, search, data collection, and data analysis drew on two analytical frameworks and an external environmental analysis method borrowed from the business discipline.

The term ‘Australian packaged food sector’ in this study includes food processing/manufacturing and supermarket retailing in which the food manufacturing and supermarket industries operate to produce and sell packaged foods. The supermarket industry refers to full-service supermarkets – four supermarkets account for 80% of revenue, of which two dominate the market.^41^ In contrast, food processing is diverse, and as defined in the Australian and New Zealand Standard Industrial Classification System (ANZSIC)^42^ includes manufacturing and processing ‘groups,’ (eg, meat and meat product and bakery product manufacturing). Within each group there are industry ‘classes’ (eg, the cured meat and smallgoods manufacturing class is a part of the meat and meat product ‘group’). To explore how external and competitive food system drivers can be mediated by the characteristics of an industry, we selected a sample of five ANZSIC classes that were illustrative of food manufacturing industries producing products in the major food groups known to be leading dietary sources of salt (eg, meat products such as processed meat, and bread and cereal products such as bread and pastries),^43^ and for which there was a corresponding Dialogue salt reduction target.^19^ The food manufacturing industries were: cheese (ANZSIC C1133(a)),^44^ cured meat and smallgoods (processed meats) (ANZSIC C1113),^45^ snack food (ANZSIC C1191),^46^ cakes and pastry (ANZSIC C1172),^47^ and bread manufacturing (ANZSIC C1171).^48^

###  The Analytical Framework

We derived our analytical framework from two established business frameworks; PEST and Michael Porter’s Five Forces.^31-33,49,50^ Traditionally these frameworks have been used as part of an external and competitive analysis of commercial^51^ and non-commercial organisations^52-54^ and are included in standard business school texts.^51^ This type of analysis seeks to identify and appraise the evidence in order to identify threats and opportunities originating from institutions and people outside the organisation that affect its operations in a given sector or industry.^33,55^

**Figure 1:**
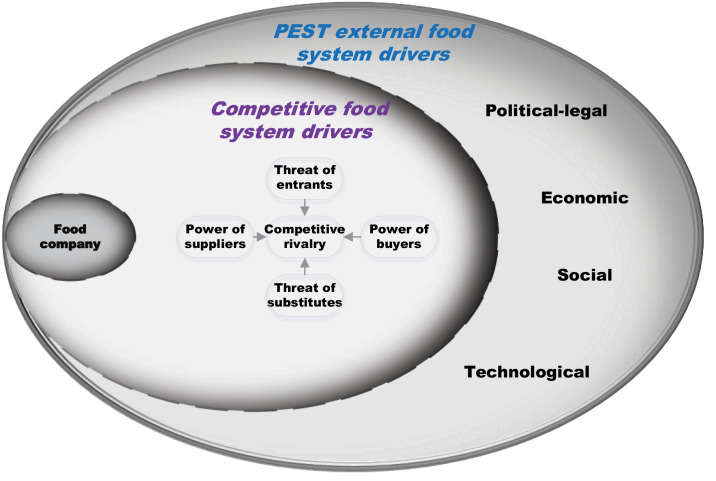
The Analytical Framework: PEST External Food System Drivers, and Porter’s Five Forces Competitive Food System Drivers. Abbreviation: PEST, political-legal, economic, social, technological.

In this study we combined the PEST and Porter’s Five Forces frameworks into one analytical framework, to show the PEST external drivers, and competitive drivers of the food system Figure 1. We used this adaption to identify the key external and competitive drivers most relevant to the Australian packaged food sector, to analyse how these might impact the willingness of food companies to voluntarily reduce salt in packaged foods and identify any implications for similar policies aiming to change the food environment.

The PEST external food system drivers are listed in the outer ring (see Supplementary file 2 for further detail). Briefly, the political-legal drivers refer to the role of the state and its stability, other political forces such as the power of stakeholder groups, the political ideology and ensuing national and international laws and regulations.^51,56^ Understanding the potential of these drivers to affect voluntary salt reduction requires knowledge of government philosophy as it influences regulation and policy which in turn impact upon business decision-making. The economic drivers are a broad group of economic factors that have a direct influence on the ability of a business to realise sufficient profit to remain competitive and access future funding.^57^ Examples include input costs to manufacturing and the economic growth rate.^51,56^ An understanding of economic drivers can elucidate the nature of the economy and competition.^33^ Social drivers are the cultural and demographic factors that include values, beliefs, lifestyle preference, and socio-economic status.^51,56^ Information of these drivers in relation to salt reduction can provide insights into consumer behaviour.^31,58^ Technological drivers relate to the introduction and adoption of technologies, and includes manufacturing, distribution and transport systems, research and development.^51,56^ These drivers can variously influence the development of new products, processes and materials for the production and sale of packaged food, and early adoption and translation of new technologies into outputs can confer a competitive advantage.^33^ The influence of one or more external food system drivers on a given industry is highly contextual and will vary over time.

A framework to identify the competitive food system drivers – Porters Five Competitive Forces framework^32,49^ is shown in the middle ring. Porter’s Five Forces is a way to analyse the competitiveness of an industry (see Supplementary file 2). Competitive rivalry refers to the intensity of competition in an industry where businesses are in direct competition. It is typically greatest when industry growth is modest, there are few differences between the products or services of competing companies, and intense competition may limit the attractiveness to invest. Rivalry is mediated by the bargaining power of buyers and suppliers, threat of entrants and substitutes. Where the power of buyers or customers can force down prices and dictate quality. For example, if there are only a few buyers buyer power is especially high, in which case buyers can force down prices particularly if it is easy for a buyer to switch between suppliers. Conversely, powerful suppliers can force prices up. The threat of entrants describes how easily a new business can enter an industry, if the barriers to entry are low, new entrants can increase competition by weakening the position and outlook of the existing businesses. The threat of substitutes refers to how easily a buyer can switch between products of comparable quality and function sold in the same industry or from another industry (eg, there are many brands of bread to choose from, but buying a pre-made sandwich from a café can be substituted for bread).

 Last, the internal factors (eg, size, scale, power and influence, resources, strategy) of each business operating within the sampled industries are not part of this study. However, for completeness, the position of the food company is shown here to depict the complexity and interconnectedness of the external and competitive food system drivers as they may influence decisions by a business at any point in time.

###  The Steps of an External Environmental Analysis – Application to This Study

As shown in Table 1, we adapted an external environmental analysis method used in strategic analysis, as the basis for our applied policy analysis.^33^Table 1 lists the 4 steps (scanning, monitoring, forecasting, assessing) used iteratively to conduct an external environmental analysis as part of a strategic analysis^33^ and our adaptation. In Step 1 *Scanning*: we iteratively searched the academic and grey literature to identify the key food system drivers most relevant to the Australian packaged food sector (2013-2016). During this time, the Australian Government purported to be pursuing salt reduction through voluntary approaches – via the Dialogue.^19^ We developed a set of key search words (see Table 2) which were informed by the analytical framework. Key data sources included multidisciplinary databases, electronic and print media for industry newsletters – often via subscription (eg, Australian Food News,^59^ FoodNavigator-Asia.com),^60^ news and views published by academics and researchers (eg, The Conversation),^61^ intergovernmental, government and organisational websites (eg, Australian Federal Government sites). One author (HT) scanned the literature for components of the external PEST and competitive food system drivers, and hand-searched reference lists to identify additional articles of interest.^62^ Articles were included if they were published in English, included any aspect of the analytical framework and were relevant for the Australian packaged food sector for the period 2013 to 2016. As this study was an applied policy analysis there was no requirement for an in-depth appraisal of quality.

**Table 1:** Steps of an External Environmental Analysis, and Adaptation for an Applied Policy Analysis

**Step**	**Application for a Strategic Analysis **	**Application for an Applied Policy Analysis **
*Scanning*	To identify early signals of shifts in the external environment.	*Search and data collection: *To identify the different components of the main food system drivers based on the analytical framework as they relate to the Australian packaged food sector.
*Monitoring*	To understand the implications through ongoing observations of changes and trends.	*Ongoing observations and data collection: *To understand the impact of and any change in food system drivers – what, how and why.
*Forecasting*	To develop projections of the future based on monitoring.	*Ongoing exploration: *To understand how food system drivers might impact the willingness of food companies to voluntarily reduce salt in packaged foods.
*Assessing*	To determine when, how, and the relative importance of change and trends for a company strategy and management.	*Ongoing exploration: *To identify any implications for similar policy aiming to change the food environment, and interpretation of findings.

**Table 2:** Summary of the Key Data Sources, Key Search Words and Document Types

**Key Data Sources **	**Key Search Words for the External and Competitive Food System Drivers**	**Key Document Types **
**Electronic databases:** ProQuest Central EBSCO, IBIS *World*, Google Scholar **Electronic and print media:** News reports, newsletters, books **Websites:** Intergovernmental, government, other organisations (eg, non-government, industry and professional)	Public health policy, corporate social responsibility, corporate governance, regulation, select committees, shared value, food, economic performance, consumer confidence, business confidence, cost of living, economic outlook, consumer trends, health trends, lifestyle preferences, research and development, innovation, food technology, reformulation, new products, salt, sodium, food processing, industry, supermarkets, food manufacturers, competitive pressure, demand, market structure, price, power, profit, investment, concentration, market share.	Articles of any type, conference proceedings, books, business summaries and reports, economic indicators and trends, government and official reports and other publications, food industry news reports, trends, expert opinion pieces, expert discussion papers.

Data analysis was primarily conducted by the lead author (HT), who examined the documentary data for relevance to the Australian packaged food sector to determine preliminary food system drivers by deductively reviewing and categorising data based on the constructs of the analytical model. Analyses were undertaken in each of *monitoring*, *forecasting* and *assessing* (Table 1). As part of *monitoring *we made ongoing observations and continued to collect, group and analyse data over the course of the study to enhance our understanding of the impact of change and trends in food system drivers. Monitoring was a perpetual, and principally, informal process between the researchers. Through regular discussions of progress in voluntary salt reduction we discussed the key external and competitive food system drivers that had been identified and the possible implications for voluntary salt reduction. This informal approach was supported by participation in network groups (eg, professional public health association events, receipt of business and public health newsletters, and discussions with food companies). Additionally, we incorporated our preliminary observations and findings into invited presentations on voluntary salt reduction; of which two were to a business audience (2014) and involved participatory discussion, and two as conference presentations to a public health audience (2017 and 2019). Collectively the monitoring ‘tools’ provided knowledge that enhanced our understanding of food system drivers and also supported ongoing exploration through *Forecasting. Forecasting* involved checking our understanding and interpretations of how the food system drivers might impact the willingness of food companies to voluntarily reduce salt in packaged foods. This was largely aided by the business literature (eg, industry reports – IBIS *World,*^63^ and newsletters we subscribed to such as Australian Food News,^59^ and business marketing and strategy theory). The last step, *Assessing *involved the exploration of any implications for similar policy aiming to voluntarily change the food environment, and the interpretation of findings that involved exploring the preliminary implications arising from steps 2 and 3. These were documented by HT and discussed with study researchers throughout the course of this study as part of drafting the first and subsequent drafts of this manuscript.

## Results

**Figure 2:**
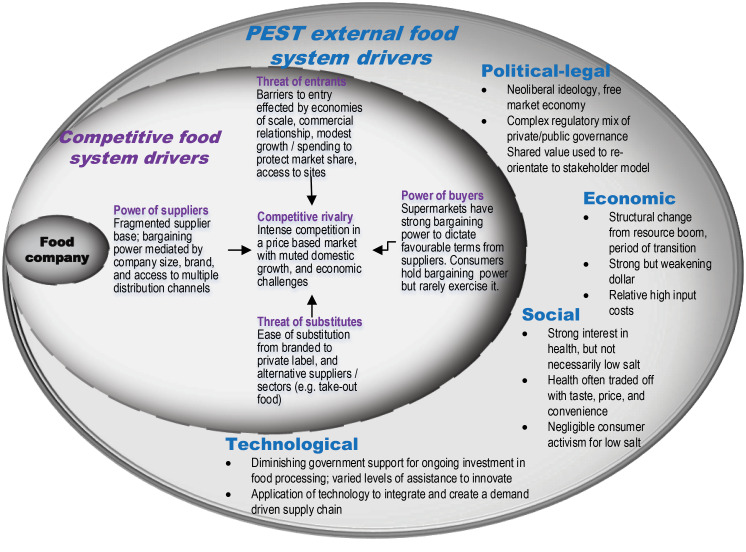
PEST and competitive food system drivers of change. Abbreviation: PEST, political-legal, economic, social, technological.

In all literatures we found relevant documentary evidence of drivers of change and identified these as food system drivers (See Figure 2). We found that as part of a complex food system, the interplay between food system drivers created an external and competitive environment unsupportive of voluntary salt reduction by food companies. Analysis by industry highlighted the highly contextual nature of food system drivers, their impact upon a given industry and the need to consider context specificity in nutrition policy aiming to change the food environment. The results are reported using the external drivers of the food system (PEST) followed by competition as a food system driver. The implications for salt reduction and other nutrition policies aiming to change the food environment are summarised in the Discussion section.

###  External Drivers of the Food System 

####  Political-Legal Food System Drivers

In addition to the state, other organisations compete for a voice in laws and regulations.^33^ Well-designed regulation is recognised as supporting the economy, reducing anti-competitive behaviour, and providing a level-playing field.^57^

The types of organisations competing to influence state regulation and policy relevant to nutrition included Australian advocacy organisations^64-66^ and the more powerful domestic food industry with a narrative of jobs, growth and investment.^67^ Though there was some evidence the Australian public supported government requiring food companies to reformulate, this was not specific to salt.^68^ We found no evidence of a grass-root societal campaign or investor led campaigns to ‘divest of salt.’

We identified a pervasive neoliberal ideology, as popularised from the work of Friedman^69^ which emphasises individual choice, personal autonomy, and a liberal market economy.^70,71^ A shareholder model of corporate governance is a characteristic of business in a liberal market economy such as Australia^72^ whereby the most important stakeholder is the shareholder, and maximising short-term shareholder value the primary marker of success.^73,74^ Though there was some evidence the shareholder model was coming under increased scrutiny^75^ the Australian law on directors’ duties provides limited guidance to directors on the scope of business actions permitted beyond those for the direct financial benefit of a company.^75,76^

The Australian federal system of government divides power between the commonwealth, state/territory, and local levels.^77^ This means that in addition to private, self-imposed, standards^78^ – often a condition of doing business^79-82^ – Australian businesses are exposed to multiple levels of state intervention affecting their operations (and profitability). Regulatory areas relevant to the production and sale of packaged food include work health and safety, environmental, employment, transport, taxation (eg, federal taxation, state and local levies), food standards and labelling, pricing, supply, trading, and competition. Food companies must comply with the relevant regulations of the industry in which they operate, meaning that the regulatory impost will vary by food manufacturing industry; we found regulation varied from light (cake and pastry)^47^ to heavy (processed meats).^45^ Nonetheless, a plethora of ‘red-tape’ – especially from duplicate and poorly designed regulation - was widely perceived by business, including food, as inefficient, costly, and an impediment to growth and investment.^83-85^

Although the regulatory burden of Australian business was analogous to that of other countries,^57^ the incumbent Prime Minister (2013-2015) pushed to remove ‘red-tape.’^86^ This, alongside a business preference for an arm’s length relationship with the state^72^ also appear to have influenced nutrition policy as exemplified by the Dialogue.^16,19^ Such ‘soft law’ approaches to government engagement with the food industry were politically more acceptable compared with ‘hard law’ alternatives.^87^ Yet, we found the Australian packaged food sector met few of the industry-level factors required for successful self-regulation including product homogeneity, concentration and tangible economic benefits to businesses from the adoption of self-regulation.^88^ Whilst we found product homogeneity and concentration were more likely to be met in the food manufacturing industries (where a specific group of companies or businesses operate in a similar area)^30^ there was little evidence of tangible economic benefits to business from salt reduction.

####  Economic Food System Drivers

Our focus here is on the drivers in the domestic economy, but we acknowledge the substantive influence of the global economy.^33^ We identified several economic drivers that created a challenging environment for food companies who needed to ‘ *put goods on the shelf at a price that works for producers, manufacturers and consumers*.’^89^ Australia was generally regarded as a small scale but geographically large^90^ and expensive country in which to manufacture food with high costs of labour and utilities.^84,89,91^ The Australian economy was also undergoing a structural change with the end of the mining investment boom from a protracted 20-year period of strong income growth to one of slower growth, weakening terms of trade, higher unemployment, sluggish nominal wage growth, and, slowing of the standard of living (income-cost of living).^57,92,93^

Although Australia is a net food exporter, the strong but weakening Australian dollar^57^ eroded the competitiveness of Australian food overseas and increased that of food imports (eg, in supermarkets).^41,94^ While imports of inputs for domestic processing (eg, additives, packaging materials)^84,94,95^ were also cheaper, salt remained one of the cheapest (and most versatile ingredients).^96^

####  Social Food System Drivers

In addition to the food environment, individual and social factors that affect consumer behaviour were found to include socioeconomic status, culture, knowledge, beliefs, and attitudes about food and food labelling.^97^ Whilst social factors contribute to social inequalities, teasing out influences on food choice is highly complex^58,97^ though a higher socioeconomic status is generally associated with a healthier diet.^98^

Despite high consumer support across the socioeconomic spectrum for the reduction of added salt,^68^ salt was found to be an attribute few consumers actively looked for on the product label.^68,99,100^ Additionally, difficulty in interpreting nutrition labels may also have different effects across socioeconomic groups.^101^ Health literacy was cited as one possible reason for people of lower socioeconomic status being less aware and unlikely to use the Australian Government led Health Star Rating System – a way of comparing the nutrient content of similar packaged foods rated from 0.5 to 5 stars.^102^

Effective marketing and promotional strategies are underpinned by a thorough analysis of the most relevant drivers for the products/services a business provides to its customers^31,103^ – and in the case of food often to the detriment of public health.^104^ Health was found to be a product attribute of increasing value to consumers;^105^ but at the point of purchase often traded off against taste, convenience, and price.^90,106,107^ With respect to specific ‘health concerns,’ low salt was frequently weaker compared to the push for low-fat or low-sugar.^45,47,48,108-110^ The salience of salt to consumers as a product attribute was found to differ between industries (eg, lower salt was a strong product attribute in snack food).^46^ Similarly, consumer acceptability of products with a lower salt content was found to differ between foods produced across the food manufacturing industries (eg, a reduction of up to only 37% in bread but as high as 67% in processed meat was possible without compromising consumer acceptability).^111^

####  Technological Food System Drivers

Industry and government policies promoting investment in infrastructure and training (eg, in food technology) was reported to be fundamental for the identification and translation of technologies in product, production and packaging development to drive growth, efficiency, and ensure a reliable, efficient supply of safe and nutritious food.^90,95,112,113^ However, findings indicate industry investment in technology was relatively low at the time of this study; net capital investment in food manufacturing had declined^114^ and concerns about diminishing government support for investment, particularly in skills^84,112^ were reported to risk the competitiveness of Australian-based companies at a time when some food companies were off-shoring research and development. The technical challenge to reduce salt differs with the food type. In addition to physical and sensory attributes such as taste and texture, salt reduction has potential knock-on effects for processing, packaging, shelf-life, food safety and preparation.^99,106,115^ Reformulation requires a business to invest its resources; it was noted that sharing innovation within an industry was mediated (at least in the case of processed meats)^45^ by the number of food companies and their relative market power within an industry.

####  Competition as a Food System Driver

Competition as a driver of change refers to the rivalry between businesses offering products that are broadly similar; competitive advantage is secured when a business provides products that satisfy consumer needs better than their competitors, meaning that businesses must consider consumer needs and competitor actions within any given industry.^31^ In our study industries include the supermarket and food processing industries in which food companies who retail or primarily manufacture similar products compete. For example, food companies in the bread industry will compete with each other but not necessarily compete with food companies in the cheese industry. The structure of an industry includes the number of competing companies and market share concentration, which is one factor that influences competition. In many instances between 2-3 food companies were found to dominate any given industry.^84^ Of the food manufacturing industries we reviewed, with the exception of cake and pastry manufacturing which was low,^47^ industry concentration in 4 was medium^44-46,48^ (ie, 4 food companies account for 40%-70% of industry revenue) and high in the supermarket industry^41,116,117^(ie, 4 supermarkets account for >70% of industry revenue).

We found the level of competition (as defined by IBIS *World*^63^ – see Supplementary file 3 for the IBIS *World* criteria used to define the level of competition) was high in the supermarket^41^ and food manufacturing industries^44,46-48^ with the exception of processed meats^45^ which was medium. The supermarket industry,^41^ cheese,^44^ processed meats,^45^ and bread^48^ food manufacturing industries were also mature, whilst the cake and pastry food manufacturing industry^47^ was in decline, and snack food manufacture was growing.^46^ Subdued economic growth, soft consumer demand^114^ and a typically slower growth relative to the economy for mature industries^41^ were identified as likely to further intensify competition. In this situation theory^32^ posits a business may experience pressure to lower prices, and defend market share through investment in marketing and promotions.

We found the threat to market share and thus profitability of existing food companies within the five food manufacturing industries included in this study was mixed. With the exception of cake and pastry (low)^47^ and supermarkets (high)^41^ barriers to entry for the other food manufacturing industries were medium.^44-46,48^ Despite high barriers to entry in the supermarket industry, ALDI and Costco (but ALDI in particular) have proved to be a potent source of new competition in a price-based market^84^ to the incumbent full-service supermarkets (Coles and Woolworths) of similar size and offering.^117,118^ ALDI redefined the retail landscape with a low-priced but quality private-label offering^119^ forcing Coles and Woolworths to invest in their private-label range.^120,121^ More than a decade of intensified and prolonged price rivalry^84,117,122,123^ ensued with Coles and Woolworths accused of playing copy-cat strategy.^122^ The net impact was perceived as squeezing profitability throughout the Australian packaged food sector^122,124^ within the context of a challenging economic trading environment.

The introduction of the voluntary Food and Grocery Code of Conduct in 2015, was described as a “step towards levelling the playing field”^125^ between supermarkets and suppliers and particularly helpful to smaller food companies with a weak brand^118^ and/or market position.^117,118,126^ Up until this time, data sources described supermarkets striking unfavourable terms with suppliers in supply contracts and imposing marketing fees.^84,117,118^ In addition to leveraging power in marketing, product placement, shelf-allocation, and retail pricing decisions,^117^ and substituting domestically produced foods with imported and cheaper alternatives.^118^

In relation to the threat of substitution with regard to consumers as buyers we found the food service sector provided consumers with an easy substitute for home cooking and thus products usually bought from a retailer such as supermarkets could be substituted for those bought from restaurants, fast food take-away outlets or online delivery.^41,122,127^ Of the food manufacturing industries we examined we also identified substitution between products (eg, on-the-go breakfast drinks, cereal and yogurt bars) were substitutes for bread^48^ – and widely available in both retail and food service industries within the consumer food environment (eg, supermarkets, small grocery stores, convenience stores, garage forecourts, and cafes).

## Discussion

Our results show that the interplay of food system drivers has created a highly unfavourable environment for voluntary salt reduction in Australia. A dominant neoliberal paradigm, and market expectation for linear economic growth and short-term profitability reinforce a shareholder model of corporate governance and intense competition with potential to dampen profitability and constrain innovation for health. Faced with a difficult economic environment and in a price driven market there appears to be no economic driver to incentivise domestic food companies to voluntarily reduce salt. Further, a weak overall trend for salt reduction is unlikely to incentivise sufficient food companies to prioritise salt reduction across their portfolio. To support a voluntary approach, lower salt foods must be commercially viable to further both economic and public health goals. The argument that compliance with voluntary opportunities can be a means for companies to differentiate from competitors, enhance competitive advantage and potentiate profit^128,129^ did not appear to hold true for salt reduction for the period of this study. Identifying ways to change this unfavourable situation are critical if Australia is to achieve the 2025 salt reduction target.^38^ The following sections outline how key food system drivers could be harnessed to influence business behaviour in the direction of action with voluntary nutrition initiatives, as well as the implications of our findings for the design of nutrition initiatives themselves.

We identified three major themes from our analysis of external and competitive food system drivers relevant to salt reduction and potential implications for nutrition policy. First, we identified the shareholder model was coming under increased scrutiny, if the purpose of a business is perceived to *both* make a profit and deliver upon broader societal goals.^74,130-132^ More recent data indicate a continuing trend^133-135^ to adopt this position. Although our results show there is little legal incentive for food companies placing non-shareholder interests (such as salt reduction) at the heart of the business agenda, as experienced by Campbell Soup who famously reduced salt in soups for health, but lost market share and announced to investors salt was being added back.^136^ This finding aligns with a more general critique of the stakeholder model as largely failing to consider the political-legal and economic food system drivers^137^ and a somewhat undefined concept in practice.^138^ Yet, its adoption could provide an opening for the concept of shared value. Proposed by Porter, shared value is where policies and practices to increase competitiveness simultaneously improve the economic and social conditions in the communities in which they operate.^130^ Its application in the Australian packaged food sector could be to frame nutrition policy to shareholders. The United Nations Global Compact - whose mission is to provide ‘business as a force for good’ –supports companies to advance societal goals based on the SDGs,^139^ and other global food corporations have also adopted shared value.^140,141^ The incorporation of nutrition considerations into investor decision-making could be one way of applying shared value. Indeed, a recent review noted substantial scope to apply a range of investment strategies used for other areas of responsible investment to the area of nutrition.^142^ While the inclusion of nutrition-related considerations as part of investment decisions is currently extremely limited,^142^ 50 investment firms managing US$3 trillion-worth of assets have signed the Access to Nutrition Index Investor Statement, signalling a commitment to consider nutrition in their investment analyses.^143^

Second, there is an opportunity to harness the power of supermarkets and consumers. While we found the asymmetry of power in favour of the supermarket industry had negatively impacted upon the Australian packaged food sector, the Food and Grocery Code of Conduct has made some positive change to relationships between retailers and suppliers although there remains some way to go.^144^ Healthy competition benefits all stakeholders. Seen in this context, supply contracts with food manufacturers are an opportunity to promote collaboration, commitment and investment in innovation.^145,146^ For example, supermarkets could adopt in-store marketing, promotion, ranging, shelf allocation, and labelling practices that support product reformulation initiatives.

Supermarkets have demonstrated their use of market power to benefit broader consumer and societal good during the recent food system shock arising from the coronavirus disease 2019 (COVID-19) pandemic and associated responses.^147^ In response to pandemic-prompted panic-buying that placed pressure on the food distribution system that led to shortages, supermarket management identified opportunities for constructive collaboration and new supply and distribution strategies. This was facilitated in Australia by the relaxation of competition law,^148^ further highlighting the opportunity for strategic policy to maximise food system benefits. Though yet to manifest as a driver for lower salt, there is a clear opportunity to leverage off consumer health trends for nutrition policy, although this might be tempered by uncertainty in economic recovery and even higher price driven motives of consumers as one impact of the COVID-19 pandemic.^149^ Competitive advantage requires food companies providing products that meet consumer needs better than their competitors. Capturing consumer perspectives on the quality and usage of the food environment is similarly crucial for ensuring that nutrition interventions meet consumer needs. Citizen Science, used widely in the natural sciences is used infrequently in public health nutrition,^150^ yet holds tremendous potential to release the largely unrealised bargaining power of the consumer.

Third, understanding food system drivers and responsiveness can inform nutrition policy design. Our analysis, shows food companies perception of an already onerous regulatory burden could curb support of regulation – a process that involves a focused attempt to change behaviour according to defined standards or purposes, with the intention of producing a particular outcome or outcomes^151^ – whether by state or non-state actors and part explains why ‘hard law’ such as mandating salt reduction targets has generally been resisted. Overcoming this would depend upon political pressure applied to the Australian packaged food sector, and independent monitoring.^152^ The apparent absence of powerful organisations and movements advocating for nutrition policy appears to have been a contributory factor for policy inertia for salt reduction.

Exploring regulatory mechanisms^151^ would benefit future policy initiatives. In addition to the shortcomings of the Dialogue (eg, lack of monitoring, limited government leadership and Dialogue inactivity)^16^ we identified that voluntary self-regulation is conditional upon the external environment^88,153^ and is an uneasy fit in the Australian business environment. Even when external and competitive food system drivers encourage food companies to make voluntary changes, there is little evidence on whether and in what circumstances voluntary ‘soft’ forms of regulation are more cost-effective than stronger forms.^11^ Mandatory salt reformulation has been found to consistently remove larger quantities of salt from the food supply than voluntary reduction.^152^ In light of product substitution, it is evident that nutrition policy (and salt reduction) needs to consider the many ways in which the consumer interacts with their food environment. A comprehensive national nutrition policy applicable to multiple sectors and food industries, with clear implementation and accountability structures,^154^ could lessen the threat of substitutes from unhealthier alternatives and minimise any commercial risk.

We identified that the structure of an industry could be a barrier to sharing salt reduction expertise and knowledge, and could be worthy of consideration in the strategic deployment of resources to provide businesses with technological support. Opportunities might include the provision of pathways and resources to assist smaller companies – as the Scottish Government did with the provision of free, tailored recipe reformulation support^155^ or the more recently Vic Health ‘Unpack the salt, salt reformulation in Australia.’^156^

While, this applied policy analysis has focused on salt reduction, the frameworks we have adapted here could be used to analyse other food system issues to identify further enablers or barriers arising from external and competitive food system drivers to transforming the food environment. Nutrition policy at all stages of the lifecycle faces many hurdles; there is a growing awareness of the importance to better understand these drivers and their interactions.^157^ Our analysis illustrates the importance of identifying food system drivers, their interconnections and context specificity if global recommendations are implemented locally. In particular, the specific attention to competition as a food system driver in our analytical framework extends the contribution of other food system analysis approaches that have already proved valuable in public health, including value chain and supply chain analysis.^51,158,159^

The aim of the present study was to unpack key external and competitive food system drivers and how they might shape business decision-making. We acknowledge that the scope of our study included only a sub-set of food manufacturing industries, however these were illustrative of key food industries producing foods targeted for salt reduction as part of the Dialogue. The scope and design of our study precluded an analysis of the internal factors (eg, size, scale, power and influence, resources, strategy) as strengths and weaknesses of the many food companies within those industries. It is almost certain that internal factors of each business will differ and will be powerful influences on their capability and capacity to plan for and react to the opportunities and threats posed by external and competitive food system drivers.^51^ Future research could incorporate analysis at a business level to highlight the heterogeneity of food companies – their strengths and weaknesses – as part of a strengths, weaknesses, opportunities and threats analysis of the implications for salt reduction in a given industry.

We took an interdisciplinary approach in this study, identified as a critical first step to understanding complex systems.^29^ We drew on two business frameworks and a method used widely in business but unfamiliar to many public health practitioners. Translating this for a non-business audience has been challenging as has the breadth and content of data requiring an understanding of multiple disciplines. Our experience aligns with a core challenge in the political economy analysis for health; to find a robust method of analysis that can be easily learned, applied and generates usable knowledge for stakeholders.^160^ Although we relied on the authors’ knowledge of public health, business, and policy, there was the potential for knowledge gaps and bias. In using qualitative data, much from the grey literature, there was an additional risk of bias where documentary data is edited to frame a message in a particular way.^161^ However, the steps we undertook including scanning, (data search, and selection) sought to mitigate this risk by iterative collection from multiple sources over a period of time and excluding articles with a blatant bias.

 In conclusion, business models can provide meaningful insights for nutrition policy on how external and competitive food system drivers can thwart policy goals. In this case, the nuances of external food system drivers highlighted areas of focus to incentivise voluntary action and illustrated the importance of political-legal, economic and consumer strategies to reduce salt in packaged foods. Our examination of food system drivers indicates that there is an opportunity for public health actors to harness food system dynamics including ‘shared value’ and the power of supermarkets in conjunction with creative regulatory strategies to increase the likelihood of a successful nutrition policy outcome.

## Acknowledgements

 The authors would like to thank Professor Shaun Larkin, Professor of Health Policy and Financing Menzies Centre for Health Policy, the University of Sydney, and Dr. Belinda Reeve, Sydney Law School, the University of Sydney for their review of this manuscript.

An earlier version of this work was included in HTs doctoral thesis (The University of Sydney, https://ses.library.usyd.edu.au/handle/2123/8974/discover) as an unpublished chapter. Since this time the study has been substantially revised. Preliminary findings based on the PhD unpublished chapter were presented as oral abstracts at the 15th World Congress on Public Health, Adelaide, Australia (April 3-7, 2017) and International Society of Behavioural Nutrition and Physical Activity, Prague (June 4-7, 2019).

 GS is an academic partner on a healthy supermarket intervention trial that includes Australian local government and supermarket retail (IGA) collaborators. In 2018, GS led a study to benchmark the policies and commitments of food companies related to obesity prevention and nutrition.

 MC interacts regularly on a non-financial basis with multiple large corporations in the Food Processing Industry and the Quick Service Restaurant industry in Australia as a part of her work to improve the quality of the food supply.

## Ethical issues

 As this research involved only the analysis of publicly available data, institutional ethics approval was not required.

## Competing interests

 GS reports grants from National Heart Foundation of Australia, during the conduct of the study; grants from National Health and Medical Research Council, outside the submitted work. BN reports other from Discovery Ltd South Africa, other from Woolworths Ltd, other from Quantium Health, other from ALDI Australia, other from Nielsen Australia, other from Bupa Australia, other from Nestle Australia, other from Access to Nutrition Foundation, other from Choices International, outside the submitted work.

## Authors’ contributions

 HT conceived of the study and early ideas for application of business models, collected data, led the analysis, interpretation of findings and drafted the initial and subsequent versions of the manuscript. AMT and BN provided guidance in the study design, analysis, interpretation and subsequent manuscript preparation. All authors contributed to the analysis and interpretation of data, and critically revised the manuscript for important intellectual content. All co-authors contributed to the manuscript as presented here.

## Funding

 This research was first undertaken at The George Institute for Global Health, where HT was supported by a postgraduate scholarship from the National Health and Medical Research Council Australia. HT is currently at the Menzies Centre for Health Policy, supported by a joint National Health and Medical Research Council of Australia and National Heart Foundation of Australia Early Career Fellowship (APP1130871). BN is supported by a National Health and Medical Research Council of Australia Principal Research Fellowship (APP1106947). BN and GS work within a NHMRC Centre for Research Excellence in Salt Reduction (APP1117300). MC was supported by an Australian Postgraduate Award when this research was first undertaken. GS was supported by a Heart Foundation Future Leader Fellowship (102035) from the National Heart Foundation of Australia. He is also a researcher within a NHMRC Centre of Research Excellence in Food Retail Environments for Health (RE-FRESH) (APP1152968) (Australia).

## Disclaimer

 The views expressed in this article are the authors’ views and not an official position of the institutions or funders.

## Authors’ affiliations

^1^Menzies Centre for Health Policy, University of Sydney School of Public Health, Sydney, NSW, Australia. ^2^Food Policy Division, The George Institute for Global Health, The University of New South Wales, Sydney, NSW, Australia. ^3^The School of Public Health, Faculty of Medicine, Epidemiology and Biostatistics, Imperial College of Science, Technology and Medicine, London, UK. ^4^Charles Perkins Centre, University of Sydney, Sydney, NSW, Australia. ^5^The Royal Prince Alfred Hospital, Sydney, NSW, Australia. ^6^Department of Urban-Global Public Health, Rutgers School of Public Health, Newark, NJ, USA. ^7^Discipline of Marketing, University of Sydney Business School, Sydney, NSW, Australia. ^8^Global Obesity Centre, Deakin University, Geelong, VIC, Australia.

## Supplementary files


Supplementary file 1. Summary of the Australian Health and Food Dialogue Targets and Results.

Supplementary file 2. A description of Food System Drivers.

Supplementary file 3. The IBISWorld Criteria Used to Define the Level of Competition.
