# Supplementary material for: Drawing on Strategic Management Approaches to Inform Nutrition Policy Design: An Applied Policy Analysis for Salt Reduction in Packaged Foods
Source: Int J Health Policy Manag. 2020 Nov 1;10(12):896–908. doi: 10.34172/ijhpm.2020.204 (PMC9309969; doi:10.34172/ijhpm.2020.204)
Supplement: Supplementary file 1 — Summary of the Australian Health and Food Dialogue Targets and Results. [file ijhpm-10-896-s001.pdf]

Supplementary file 1. Summary of the Australian Health and Food Dialogue Targets and Results

All data in Table S1 and definitions have been sourced from: Australian Government Department of Health. Healthy Food Partnership-Food and Health Dialogue. Available from: https://www1.health.gov.au/internet/main/publishing.nsf/Content/fhd.

Table S1. Summary of the Dialogue sodium reduction targets and timeframe for action

Food Category	Sodium Reformulation Target	Timeframe for action
Bread	400mg/100g on defined bread products[1].	May 2010 – Dec 2013
Ready-to-Eat Breakfast Cereal	15% reduction on defined products that exceed 400mg/100g[2].	May 2010 – Dec 2013
Simmer Sauce	15% reduction for Asian-style simmer sauces[3] exceeding an action limit level of 680mg/100g.	Jan 2011 – Dec 2014
	15% reduction for pasta[4], Indian-style[5] and other simmer sauces exceeding the action level of 420mg/100g.	
	Seek opportunities to reduce sodium levels across simmer sauces that are significant contributors to market share, but are below the action level of 680mg/100g (Asian-style); 420mg/100g (pasta, Indian-style and other simmer sauces).	
	Aim for 15% reduction in new products versus current like benchmarks, when the benchmarks are above the action levels, and as low as practical when below action levels.	
Processed Meat[6]	Maximum sodium content of nominated bacon and ham/cured meat products to 1090mg/100g.	Jan 2011 – Dec 2013
	Maximum sodium content of nominated emulsified luncheon meats to 830mg/100g.	
Soups	Maximum sodium content of 290mg/100g across dry soup products.	Dec 2011 – Dec 2014
	Average sodium target (weighted by sales volume) of 290mg/100g and a maximum target of 300mg/100g across wet/condensed soup products.	
Savoury Pies[7]	10% reduction in sodium across wet savoury pies with sodium levels exceeding 400mg/100g.	Mar 2012 – Mar 2014
	10% reduction in sodium across dry savoury pies with sodium levels exceeding 500mg/100g.	
Potato/Corn/Extruded Snacks	Average sodium target of 550mg/100g and a maximum target of 800mg/100g across potato chip products[8].	Dec 2012 – Dec 2015
	Average sodium target of 950mg/100g and a maximum target of 1250mg/100g across extruded snack products[9].	
	Average sodium target of 850mg/100g and a maximum target of 1100mg/100g across salt and vinegar snack products[10].	

Food Category	Sodium Reformulation Target	Timeframe for action
	Average sodium target of 550mg/100g and a maximum target of 700mg/100g across cereal based snack products[11].	
Savoury Crackers	Maximum sodium content of 850mg/100g across plain crackers[12], or, 15% sodium reduction towards the maximum target for products significantly higher than the maximum.	Dec 2012 – Dec 2015
	Maximum sodium content of 1000mg/100g across flavoured crackers[13], or, 15% sodium reduction towards the maximum target for products significantly higher than the maximum.	
	Maximum sodium content of 850mg/100g across flavoured rice crackers, flavoured rice cakes and flavoured corn cakes[14] or, 15% sodium reduction towards the maximum target for products significantly higher than the maximum.	
	No target for plain rice crackers/cakes/corn cakes[15].	
Cheese	Maximum sodium target of 710mg/100g for Cheddar and Cheddar Style variety cheese products[16].	Mar 2013 – Mar 2017
	Maximum sodium target of 550mg/100g across Mozzarella Cheese products[17].	
	Maximum sodium target of 1270mg/100mg, or, a 10-15% reduction in sodium towards the maximum target for chilled processed cheese products[18] with sodium levels significantly above the agreed maximum target of 1270mg/100g.	

Definitions

[1] Defined bread products: those made by baking a yeast-leavened dough prepared from one or more cereal flours or meals and water. This includes, for example, sliced loaf breads, rolls, bagels, English muffins and fruit breads. The definition does not include value-added products such as cheese and bacon rolls.

[2] Defined ready-to-eat breakfast cereal products: plain or mixed flakes, puffed grains, processed grains, fruit/flake mixtures with or without other ingredients. Ready-to-eat cereals do not include muesli, hot cereals or wheat biscuits.

[3] Based on flavours to replicate Asian recipes. Often based on high sodium sauces (eg, soy, fish or oyster sauce) and often labelled as noodle sauce or stir-fry-sauce.

[4] Mainly tomato-based sauces, but includes pasta bakes, may also be chunky or smooth and contain other ingredients such as vegetables and/or meat. Pasta sauces can be pout-in, stir-in, cook-in or stir-thru.

[5] Based on flavours to replicate Indian receipts and often labelled as curry sauces.

[6] Defined by the Dialogue as: ready-to-eat meats (excluding pate, cooked uncured meats (eg, roast meats) and dried meats); bacon, and pasteurised sausages (including frankfurters, chorizos, cheerios etc.)

[7] Defined savouring pies: wet meat and vegetarian pies (meat/poultry only or with vegetables in 'gravy base' eg, steak and kidney, chicken and mushroom), and dry meat and vegetarian products (eg, sausage rolls, pasties, pork pie and pot pie).
[8] Defined potato chip products: thin potato slices that are generally deep fried and then flavoured using eg, salts, seasonings, herbs or spices. All flavours except salt and vinegar. (eg, thick-cut, thin-cut, crinkle-cut, sticks, deli-style, Pringles).
[9] Defined extruded snack products: starch-rich materials (eg, corn, maize, wheat, rice, potato flour) that are transformed into hot melt fluids and then expanded or puffed via an extruder to form a snack. All flavours except salt and vinegar. (eg, Cheese Puffs, Bacon Balls, Twisties, Burger Rings, Cheezels).
[10] Defined as all products that are salt and vinegar flavoured.
[11] Defined cereal-based snack products: cereal grains (eg, wheat, corn, maize kernels used to make dough, which is then sheeted to thin, uniform dimensions and cut to form the snack and fried. All flavours except salt and vinegar. (eg, corn-based snacks such as Doritos, and wholegrain snacks such as Grain Waves).
[12] Defined plain crackers (flour-based): crackers with no added flavourings. Includes pepper/grain varieties, but not those with added salt flavours. (eg, plain crackers with a flaky texture, wholegrain/wholemeal crackers, crispbreads, other varieties such as water crackers.
[13] Defined flavoured crackers (flour-based): crackers with added flavourings. Includes those with added salt flavours. (eg, savoury biscuits, flavoured crackers with flaky texture, other herb and salt varieties).
[14] Defined flavoured rice crackers/cakes/corn cakes: cakes made from puffed rice/corn, with added flavourings, crackers made from predominantly rice flour, with added flavourings, includes those with added salt flavourings and 'original' flavoured.
[15] Defined plain rice crackers/cakes/corn cakes: cakes made from puffed rice/corn, with no added flavourings, crackers made from predominantly rice flour with no added flavourings.
[16] Defined Cheddar and cheddar style variety cheese products: mild, tasty, extra tasty, and vintage cheese products.
[17] Defined Mozzarella Cheese products: low moisture mozzarella cheese products only. Fresh mozzarella and pizza cheese blends are excluded.
[18] Defined Chilled Processed Cheese products: all processed cheese products which need to be refrigerated.

Table S2. Summary of Results for the Dialogue Sodium Reduction Targets

Food Category	Market Share (%) of the companies that committed to the Dialogue target[1]	Proportion (%) of products that met the target at baseline and collection[2,3]		Mean sodium content 100/mg ± SD at baseline and collection	
		Baseline	Collection	Baseline	Collection
Bread	80%	28	86	432 ± 95	390 ± 58*
Ready-to-Eat Breakfast Cereal	60%	55	83	393 ± 256	266 ± 160*
Simmer Sauce	85%				
Asian style		41	55	820 ± 513	825 ± 566
Indian style		40	68	497 ± 157	425 ± 182
Pasta sauces		33	76	474 ± 158	380 ± 120*
Simmer sauces		25	46	492 ± 104	476 ± 127
Processed meats	95%				
Bacon		25	59	1230 ± 250	1093 ± 186**
Ham and other cured meat		47	80	1105 ± 263	1013 ± 229**
Emulsified luncheon meat		23	44	945 ±162	901 ± 144
Soups	90%				
Dry		27	78	320 ± 69	304 ± 80
Wet		75	80	295 ± 61	280 ± 67**
Savoury Pies	85%				
Wet, meat and vegetarian pies		28	51	454 ± 94	402 ± 90*
Dry, meat and vegetarian products		37	28	567 ± 116	550 ± 82
Potato/Corn/Extruded Snacks	100%				
Cereal based snacks		88	92	579 ± 190	499 ± 163**
Potato chips		93	92	602 ± 180	589 ± 157
Extruded snacks		96	94	1064 ± 195	907 ± 227**
Salt and vinegar snacks		53	78	1045 ± 256	928 ± 269
Savoury Crackers	80%				
Flavoured (flour based)		72	79	898 ± 259	840 ± 218
Plain (flour based)		77	87	746 ± 302	630 ± 198*
Rice crackers, cakes, corn-cakes, flavoured		70	76	665 ± 323	687 ± 322
Cheese	80%				
Cheddar and cheddar style		84	86	649 ± 56	654 ± 50
Low moisture Mozzarella		63	68	522 ± 120	548 ± 105

Food Category	Market Share (%) of the companies that committed to the Dialogue target[1]	Proportion (%) of products that met the target at baseline and collection[2,3]		Mean sodium content 100/mg ± SD at baseline and collection	
Processed chilled		37	43	1300 ± 358	1341 ± 290

[1] Data sourced from: Australian Government Department of Health. Healthy Food Parternship-Food and Health Dialogue. Available from: https://www1.health.gov.au/internet/main/publishing.nsf/Content/fhd

[2] Data sourced from: The National Heart Foundation of Australia, Report on the Evaluation of the nine Food Categories for which reformulation targets were set under the Food and Health Dialogue -for submission to the Department of Health 6 May 2016. Available from: https://www1.health.gov.au/internet/main/publishing.nsf/Content/7BD47FA4705160A6CA25800C008088B9/$File/Healthy%20Food%20Partnership%20Evaluation%20Report_Heart%20Foundation.pdf

[3] The month and year for baseline data varied by food category: Bread, March 2009, Ready-to-Eat-Breakfast Cereals, Jan 2010; Simmer Sauces, and Processed Meats, May 2010; Soups, Feb 2011; Savoury Pies, and Cheese, May 2011; and, Potato, Corn, and Extruded Snacks, and Savoury Crackers, May 2012. The month and year for collection data varied by food category; Bread, Aug-Sep 2015; Ready-to-Eat-Breakfast Cereals, and Processed Meats, Apr-May 2015; Simmer Sauces, and Soups, Jun-Jul 2015; Savoury Pies, Sep-Oct 2015; Potato, Corn, and Extruded Snacks, Oct 2015; Savoury Crackers, Jul-Aug 2015; and, Cheese, Sep 2015. Note: the timeframe (start and end) of the Dialogue targets does not always align with the date of the baseline and collection data.

* significantly different to baseline ($P < .001$).

** significantly different to baseline ($P < .05$)
