# Supplementary material for: Drawing on Strategic Management Approaches to Inform Nutrition Policy Design: An Applied Policy Analysis for Salt Reduction in Packaged Foods
Source: Int J Health Policy Manag. 2020 Nov 1;10(12):896–908. doi: 10.34172/ijhpm.2020.204 (PMC9309969; doi:10.34172/ijhpm.2020.204)
Supplement: Supplementary file 2 — A description of Food System Drivers. [file ijhpm-10-896-s002.pdf]

Supplementary file 2. A description of Food System Drivers

Political-legal, economic, social, and technological (PEST) external drivers

- ***Political-legal drivers*** refers to the role of the state and its stability, other political forces, the political ideology and ensuing national and international laws and regulations, and includes: political ideology, governance structures laws and regulations in policies such as; trade agreements; competition corporate law; food regulation; and, the power of stakeholder groups (eg, non-governmental, international health organisations, food industry associations, consumer organisations, and concerned media).
- ***Economic drivers*** refers to a broad group of economic factors and includes: the real exchange rate; economic growth rate; consumer confidence; unemployment rate, interest rate; inflation rate; and, input costs to manufacturing (worldwide commodity prices, national utility prices, cost of fuel, and labour rates).
- ***Social drivers*** refers to cultural and demographic factors and includes: cultural values; social norms, attitudes and lifestyle preferences; population growth; level of education; distribution of wealth; age distribution; household composition; ethnicity; class; and, gender.
- ***Technological drivers*** refers to the introduction and adoption of technologies and includes: manufacturing, distribution and transport systems; materials handling; marketing and sales techniques; information systems and devices; and research and development.

Competitive drivers

Our description of the competitive drivers is technically referred to in the business literature as Michael Porters Five Competitive Forces.

- ***Power of buyers*** refers to the bargaining power of the supplier. Buyers may be 'business buyers' (as in the case of supermarkets buying from food manufacturers) or the 'ultimate consumers' (buying from the supermarket) and the influence of each can vary markedly. Few buyers relative to suppliers' means that it is easier for a buyer to force down prices, dictate quality, and take most of the profit. Buyer power is especially high if it is easy for the buyer to switch between suppliers or a buyer starts to make the product they are buying ('backward vertical integration').

- ***Power of suppliers*** is the opposite of buyer power. Powerful suppliers can force prices up, limit quality improvements, and push costs on to buyers.

- ***Threat of entrants*** refers to how difficult it is for a new company to enter an industry. Where entry is difficult existing companies are most likely to benefit because fewer competitors can

mean less competition. To enter an industry and be successful, a new entrant must overcome regulatory hurdles, find access to suppliers and buyers, and be able to configure an efficient supply chain. They must also offer a product or brand that consumers perceive as being different or of better value than the current offerings.

- ***Threat of substitutes*** refers to how difficult it is for buyers to switch between products that offer comparable quality and function but are sold by different industries. Low switching costs can make it difficult for companies to increase price which can dampen profitability and reduce spend available for product development.

- ***Competitive rivalry*** refers to the intensity of competition in an industry and is mediated by the bargaining power of buyers and suppliers, threat of entrants and substitutes. Competitors of a similar size often compete intensely for sales. If entry to the industry is easy then rivalry is intensified. Competition is typically greatest when industry growth is modest and there are few differences between the offerings of competing companies. Intense competition may put downward pressure on price and paradoxically limit companies' opportunities to innovate, improve and attractiveness to invest.

Note: The description of the PEST food system drivers is taken from: Johnson G, Scholes K & Whittington R, *Exploring Corporate Strategy: Text and Cases.* 8th ed. Essex, UK; Pearson Education Limited; 2008, and Viljoen J & Dann S, *Strategic Management.* 4th ed. Frenchs Forest, Australia; Prentice Hall; 2003. The description of Michael Porters Five Competitive Forces is taken from: Johnson G, Scholes K & Whittington R, *Exploring Corporate Strategy: Text and Cases.* 8th ed. Essex, UK; Pearson Education Limited; 2008, and Viljoen J & Dann S, *Strategic Management.* 4th ed. Frenchs Forest, Australia; Prentice Hall; 2003, Porter M, How Competitive Forces Shape Strategy. *Harvard Bus Rev*. 1979;57:137-145, Porter M, The Five Competitive Forces that Shape Strategy. *Harvard Bus Rev.* 2008;86:58-77, and Kotler P, Brown L, Adam S, Armstrong G. *Marketing*. 6th ed. Sydney, Australia: Pearson/Prentice Hall; 2004.
