# Supplementary material for: Drawing on Strategic Management Approaches to Inform Nutrition Policy Design: An Applied Policy Analysis for Salt Reduction in Packaged Foods
Source: Int J Health Policy Manag. 2020 Nov 1;10(12):896–908. doi: 10.34172/ijhpm.2020.204 (PMC9309969; doi:10.34172/ijhpm.2020.204)
Supplement: Supplementary file 3 — The IBISWorld Criteria Used to Define the Level of Competition. [file ijhpm-10-896-s003.pdf]

Supplementary file 3. The IBIS*World* Criteria Used to Define the Level of Competition

The factors IBIS*World* take into account to determine the competitive landscape within an industry. These factors have been taken from: IBIS*World* as outlined in the IBIS*World* industry reports used throughout this study (Cheese, Cured Meat and Smallgoods, Snack Food, Cake and Pastry, and Bread processing/manufacturing).

.

- **Market share concentration** is considered high if the top four players account for more than 70% of industry revenue. Medium is 40% to 70%. Low is less than 40%.
- **Key success factors** differ by each industry (and/or business) and may include factors such as economies of scale, control of distribution arrangements, and supply contracts in place for key inputs.
- **Cost structure** (eg, often larger manufacturers have a lower per-unit production costs due to economies of scale). Cost structures vary with each business, an industry cost structure is an estimated average of all companies in that sector.
    - IBIS consider profit, purchases, wages, depreciation, and other expenses (eg, promotional, research and development costs) as a proportion of revenue.
- Also considered are **internal competition** (eg, price, product differentiation, and distribution networks) and **external competition** (eg, other suppliers of the product, in the case of bread this might be in-store bakeries, hot bread shops, or artisanal bakeries).
- **Barriers to entry** (the ease with which a new company can enter a market) high barriers mean it is difficult, and low barriers mean it is easier.
- **Industry globalisation** is the extent of involvement in international operations – including international trade (export offer growth opportunities and import competition whereby foreign producers satisfy domestic demand that local firms would otherwise supply).
